# Angiogenesis in Malignant Thyroid Tumors

**DOI:** 10.4021/wjon263e

**Published:** 2011-01-01

**Authors:** Matvey Vladimir Sprindzuk

**Affiliations:** United Institute of Informatics Problems, National Academy of Sciences of Belarus, Minsk. Belarus, 220040, Minsk, Bogdanovicha lane, 112/38. Email: sprindzuk@yahoo.com, sprindzuk@tut.by

**Keywords:** Thyroid cancer, Angiogenesis, Lymhangiogenesis, Malignancy, Tumor expansion, Metastasis, Vasculogenesis, Endothelium

## Abstract

It is well known that radiation significantly impacts the morbidity of thyroid cancer and that is why Belarus has the highest incidence of the malignancy. Author describes statistical data, classification of angiogenesis, and typical pathological features of malignant thyroid diseases with regard to the vascular network.

## Introduction

**Figure 1:**
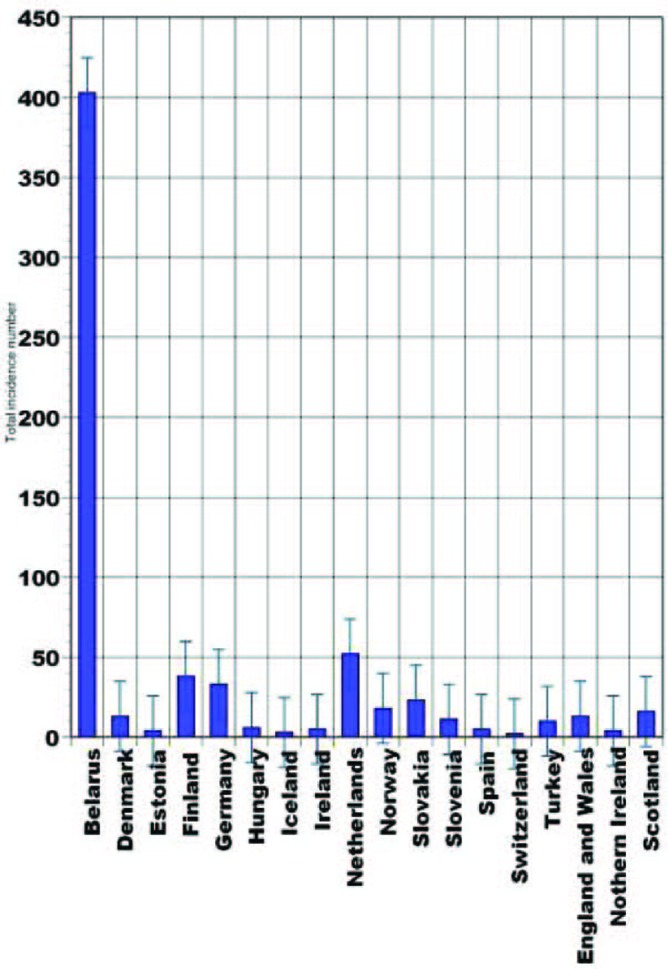
Pediatric thyroid cancer statistical data (1993 - 1997) [2].

As many as 246,347 cancers have been diagnosed during year 2007 in UK (United Kingdom) [1]. In 2006, 1,933 British people revealed thyroid cancer. Thyroid cancer is within the top 20 most common cancers for UK females (number 18), with 1,421 new cases diagnosed in 2006. This compares to 512 cases in males, giving a male:female ratio of 1:3. It has been estimated that the lifetime risk of developing thyroid cancer is 1 in 842 for men and 1 in 324 for women in the UK. These were calculated on February 2009 using incidence and mortality data for year 2001 - 2005. Thyroid cancer is rare in children, while in adults the incidence rates rise steadily with age. Although the rates are highest in the over 75s, there is a substantial number of cases at younger adult age. Almost half (48%) of all cases occur in people aged less than 50 years. In the UK the age-standardized incidence rates have increased from 1.8 to 2.9 per 100,000 population between 1993 and 2006. There has been a larger increase in female incidence rates, from 2.4 to 4.2 per 100,000 population. The highest rates for thyroid cancer in the world occur in Northern America, where the female age-standardized rate is 8.1 per 100,000 females, compared with 1.4 per 100,000 females in Western Africa. Incidence is low in all parts of Africa. The registered case number of pediatric thyroid cancers in Belarus during 1993 - 1997 was 403, and 317 of them occurred in children aged 10 - 14. Current statistics are not published yet and the expected numbers are unknown (Fig. 1). The second leading place in that epidemiologic cohort belongs to Netherlands, with 52 cases (29 persons in 15 - 19 age groups). During the described time period no juvenile thyroid cancer was reported in Switzerland [2].

Women appear to have at least twice the risk of men for clinically apparent (thyroid) cancers at a given exposure. Data suggesting that children are more susceptible than adults warrant a 50% reduction in risk coefficients when estimates derived for people less than or equal to 18 years at exposure are applied to a population of adults. For the calculation of risks of fatal thyroid cancer, current levels of medical diagnosis and care are assumed, and a maximum of 10% of the clinically evident radiation-induced thyroid cancers are expected to be lethal. After exposure to external irradiation, the projected overall lifetime incidence of fatal thyroid cancer would be 7.5 cases per 0.01Gy (Gray) absorbed dose to the thyroid in a general population of one million persons. Ethnic background was found to influence the risk of radiation-induced thyroid cancer, for example, the relative risk for Jews compared to non-Jews was about 3.5 after adjusting for gender, time since exposure, and dose. It is known since 1930 that capillaries are radiosensitive structures and Ahmad [3] et al (2007) showed that ionizing radiation decreases capillary-like structure formation by endothelial cells *in vitro*. To my knowledge, no studies were performed exploring the patterns of angiogenesis in thyroid tissues exposed to ionizing radiation and toxic agents.

Тhere are no published data from the human studies providing the description of angiogenesis patterns of every histological entity mentioned. Moreover, there is practically no information describing morphology of angiogenesis in mouse thyroid cancer models [4].

## Definitions of Angiogenesis and Its Classification

Despite existing thousands of articles, monographs and research reports describing practically all the facets of angiogenesis, little information is dedicated to the classification of angiogenesis and angiogenic morphologic patterns.

In the majority of research papers, angiogenesis is defined as the development of new vessels from the already existing vasculature, capillaries. Angiogenesis, which is essential for tumor growth and progression, does not involve a single pathway, but is a complex of many factors and signal transduction systems [5]. Endothelial cells are the source of new blood vessels, and they have a remarkable ability to migrate, proliferate, and differentiate [6].

Several distinguishing morphological and pathological characteristics were described for the tumoral vasculature versus normal blood supply in respective tissues. Malignant vessels show increased vessel tortuosity and variable vessel diameter, poorly developed and fragile vessel walls, variable flow rates leading to micro-regional tumor hypoxia, increased interstitial pressure within the tumor and increased vessel permeability, and poor connections between pericytes and endothelial cells, which demonstrate irregularly shaped endothelial cells and basement membrane as well as lack of lymphatic drainage and of vascular smooth muscle. It is assumed that increased expression of vascular endothelial factor, a potent angiogenesis stimulator, is characteristic of differentiated thyroid cancers and is associated with increased growth, progression, and invasiveness of the tumor and with decreased recurrence-free survival [7]. Hsiаo [8] et al (2005) found that the K2578 C/A SNP (single-nucleotide polymorphism) in the promoter region of the VEGF (vascular endothelial growth factor) gene may predispose the risk of development of thyroid cancer and regional lymph node metastasis. Their data also suggested a sex-specific effect and the idea that males are under stronger genetic influence than females.

**Figure 2:**
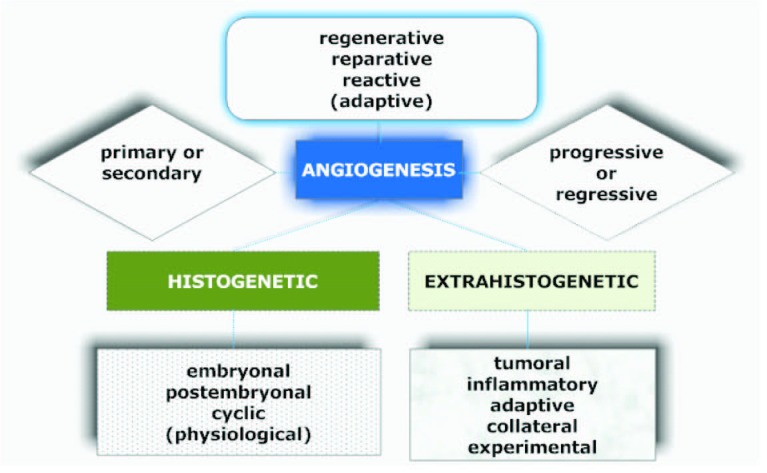
Graphical description of the classification of angiogenesis described by Kupriyanov et al [9].

**Figure 3:**
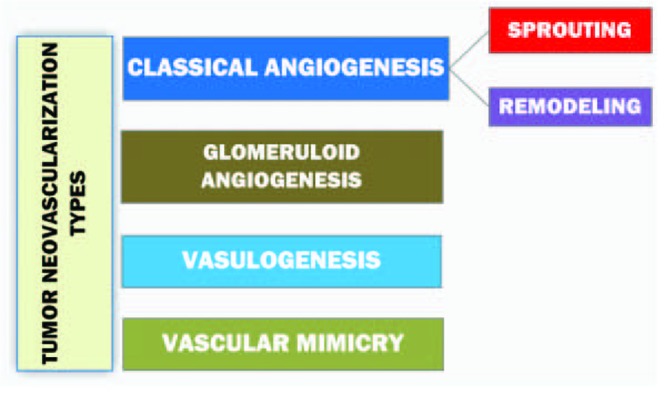
Diagram representing the classification of tumor neovascularization [10].

Informative and comprehensive classifications of angiogenesis and the neovascularization types аre depicted on Figure 2 and 3.

Sprouting as the subtype of a classical angiogenesis is the expansive outgrowth of the vascular network. Remodeling refers to the rebuild of the vascular net and intussusceptive vascular growth. The term ‘vasculogenesis’ is the *de novo* development of vessels from the endothelial cell progenitors. Glomeruloid angiogenesis is a formation of vascular structures that are similar to the renal glomeruli. Vascular mimicry is the tumoral ability to form capillary network composed of neoplasm cells without blood and lymphatic capillaries in the content of these tubular structures [10].

## The Role of Thyroid Hormone in the Development of Vasculature

The rich vascular net of the thyroid was mentioned in research article by Bradley [11] (1896) in the description of hemorrhagic cysts of thyroid gland.

Cunningham (1898), investigating the pharmacology of thyroid extracts on animal models, pointed at the fact that thyroid hormones might have a significant impact on vascularization in tissues other than the thyroid gland itself [12]. In the heart and other tissues, thyroid hormone has well-documented effects on angiogenesis. Mechanistically, most of these effects are initiated at the integrin receptor for the hormone on endothelial and vascular smooth muscle cells and reflect interfaces between non-genomic and genomic mechanisms invoked by the hormone. While physiologic concentrations of T3, T4 and certain other analogues support angiogenesis in normal tissues, it is speculated that they may be supplementally useful in certain local settings of tissue ischemia [13]. Patel [14] et al (2003) isolated a human endothelial cell strain, confirmed by Tie-2 and factor VIII-related antigen expression and NO (nitric oxide) release in response to VEGF. These cells respond to paracrine FGF-2 (fibroblast growth factor), and VEGF, though a less potent mitogen, was able to increase FGFR1 (fibroblast growth factor receptor) expression. The cells also respond to the paracrine antiangiogenic factor TSP-1 (thrombospondin) and to angiostatin generated from the plasminogen by the action of thyroid follicular cell-conditioned medium. This, and the observation that TSH (thyroid stimulatory hormone) and thyroid hormone had no apparent effect on thyroid endothelial cells, according to the researchers, suggest that angiogenesis observed during goitrogenesis is under the control of TSH-induced paracrine factors.

In 2006, Yamada [15], Emiko et al demonstrated for the first time that iodide at high concentration decreases the expression of the angiogenic factors VEGF-A, VEGF-B, and PGF (platelet growth factor), accompanied by an increase in the expression of possible antiangiogenic factors. These proangiogenic and antiangiogenic factors may at least partly account for the iodide-induced decrease in thyroid blood flow.

Zhang [16] et al (2009) showed that thyroid hormone has a substantial impact on vasculature development in the brain. Pathologically altered vascularization could, therefore, be a contributing factor to the neurologic deficits induced by thyroid hormone deficiency.

Interestingly, T4 should not be used for pro-angiogenic intent in vessels because it causes platelet agglutination. In studies of a hind limb ischemia model in intact rabbit, thyroid hormone administration induces new blood vessel formation. Thus, evidence from the intact animals suggests that circulating thyroid hormone supports angiogenesis [17].

## Essentials of Pathological Mechanisms of Angiogenesis in Thyroid Gland

According to Ramsden [18] (2000), increased vascularity in the thyroid can occur in hyperplastic goiter, Graves' disease and cancer, and may be associated with a vascular hum because of increased blood flow. In cancers of the thyroid, MVD (microvessel density) has been shown to correlate with disease-free survival in papillary carcinoma of the thyroid and intrathyroid tumor spread in follicular carcinoma. Interestingly, in experimental induction of goiter by low iodine and thiouracil in rats, Wollman et al (1978) showed that the capillaries within the thyroid clearly enlarged within 3 days of treatment, and by 20 days, they surrounded the follicles with a continuous endothelial sheet. There were both fusion of capillaries and mitosis of endothelial cells. There was no change in blood vessel morphology or number in nearby extrathyroidal tissue, including the parathyroids [18]. It is hypothesized that patterns for tumor behavior and metastasic spread vary according to tumor type, and whether differences in the angiogenic or lymphangiogenic phenotype influence the route for tumor metastases or determine a more aggressive behavior has not been fully explored [19]. It is discovered that integrin alphaVβ3 contains a cell surface receptor site for thyroid hormone that is linked to activation of mitogen-activated protein kinase and induction of angiogenesis [20]. To assess the potential role of angiogenesis factors in human thyroid tumor growth and spread, Bunone [21] et al (1999) analyzed their expression by semiquantitative RT-PCR and immunohistochemistry in normal thyroid tissues, benign lesions, and different thyroid carcinomas. Compared to normal tissues, in thyroid neoplasias researchers observed a consistent increase in VEGF, VEGF-C, angiopoietin-2 and in their tyrosine kinase receptors KDR (tyrosine kinase receptor), Flt-4 (Fms-related tyrosine kinase 4), and Tek (tyrosine kinase). In particular, researchers detected the overexpression of angiopoietin-2 and VEGF in thyroid tumor progression from a prevascular to a vascular phase. In fact, they found a strong association between tumor size and high levels of VEGF and angiopoietin-2. Furthermore, results of that study show an increased expression of VEGF-C in lymph node invasive thyroid tumors and, on the other hand, a decrease of thrombospondin-1, an angioinhibitory factor, in thyroid malignancies capable of hematic spread. These data suggest that, in human thyroid tumors, angiogenesis factors seem involved in neoplastic growth and aggressiveness. Moreover, the discovered findings are in keeping with a hypothesis that in the presence of VEGF, angiopoietin-2 may collaborate at the front of invading vascular sprouts, serving as an initial angiogenic signal that accompanies tumor growth [21].

Gerard [22], Anne-Catherine et al (2009) proposed that an inverse relationship exists between the expansion of the thyroid microvasculature and the local availability of iodine. This microvascular trace element-dependent regulation is unique and contributes to keep steady the iodide delivery to the thyroid. Signals involved in this regulation, such as VEGF-A, originate from the thyrocytes as early TSH-independent responses to iodide scarcity. The question raised in this paper is how thyrocytes, facing an acute drop in intracellular stores of iodine, generate angiogenic signals acting on adjacent capillaries. Using *in vitro* models of rat and human thyroid cells, researchers showed for the first time that the deficit in iodine is related to the release of VEGF-A via a reactive oxygen species/hypoxia-inducible factor-1-dependent pathway.

## Thyroid Cancer: General Experience in Angiogenesis Investigation

VEGF was initially discovered as a tumor-derived factor, which increased microvascular permeability. Subsequently, the protein was found to exhibit mitogenic effects exclusively on endothelial cells. In normal thyroids, VEGF was found to be present within the follicular cells and shown to be secreted in response to thyrotropin from a thyroid cancer line *in vitro*. VEGF was also elevated within goiters and, in the FRTL-5 (Fischer rat thyroid cell line), VEGF was found to significantly reduce the ability of TSH to increase 125 I uptake [14]. It is now clearly evident that VEGF-C is associated with the lymphatic tumor spreading. VEGF seems to be a significant component of the regulation of angiogenesis within the thyroid, and alteration in intrathyroid expression of VEGF is seen in many thyroid pathologies. On current understanding, VEGF is a central factor in angiogenesis in the thyroid gland, as indicated by the alterations in VEGF concentrations in many pathological conditions of the thyroid. Many other factors are involved, although some of these may act through regulation of VEGF expression in follicular cells. TSH is an important regulator of VEGF, although the role of factors important in other tissues, such as hypoxia, is not known in the thyroid. The thyroid is an excellent model for the integrated control of angiogenesis, because of the vascularity of the thyroid gland, and its capacity to increase its blood flow in disease [18].

TSH has been shown to induce VEGF expression in several thyroid carcinoma cell lines [23].

Luboshitzky [24] and Dharan (2004) demonstrated the utility of CD34 immunolocalization in cell block preparations as an adjunct to fine needle aspiration (FNA) diagnosis of thyroid cancers. Researchers reported linear ‘beaded’ and ‘looped’ pattern of angiogenesis in papillary cancer, narrow, slitlike, discontinuous, sinusoidal pattern of medullary carcinoma of the thyroid and slightly convoluted ‘gaping pattern’ in benign nodular goiter.

As early as in 1996, Karl Segal [25] et al investigated 30 paraffin-embedded samples of follicular thyroid tumors obtained from the local archives. Endothelial cells were immunostained with anti von Willebrand factor, peroxidase, diaminobenzidine and counterstained with Carazzi’s hematoxylin with a consequent standard washing procedures. A marginal difference was noted in the degree of vascularity between the follicular thyroid adenomas and the follicular thyroid carcinomas; both types were relatively vascular. Eight of the 15 adenomas examined were more vascular than the other seven. The same was true for the follicular carcinomas. However, a major difference in vascularity was noted among different areas within the follicular carcinomas. Areas of follicular carcinomas adjacent to and, especially, infiltrating the capsule showed significantly increased vascularity, with a ratio of one blood vessel to two tumor cells. Tumor areas distant from the capsule had a ratio of only one vessel to 10 tumor cells on average. Areas of adenomas adjacent to the capsule did not have prominent vascularity, in contrast to the carcinomas. Their ratio of blood vessels to tumor cells was uniformly around 1 : 10. Solid areas with marked pleomorphism within the follicular carcinomas, suggesting a higher degree of malignancy, again showed a higher ratio of vascularity than areas with no marked pleomorphism or solid formation. A 1 : 2 blood vessel tumor cell ratio was noticed in the pleomorphic sites and solid areas, whereas a much lower ratio 1 : 10 was observed in other zones. In comparing the degree of vascularity between follicular adenomas and adenocarcinomas, no significant differences were found. However, a definite difference in vascularization was noted in different areas within the follicular carcinomas. Researchers showed that the more malignant appearing areas, as indicated by pleomorphism and solidity, had a higher rate of vascularization. Areas of tumor adjacent to or penetrating the capsule were also characterized by high vascularity. Thus, although vascularity did not seem to be a distinguishing feature of follicular carcinoma, the higher vascularity in the more malignant areas of the tumor suggests that vascularity may indeed play a role in tumor aggression. The presence of high vascularity in the pericapsular area also suggests that vascularity may be important in tumor potential for extracapsular extension and expansion.

Turner [26] et al (2003) reviewed the published articles reporting the data where the principal objectives of investigation were the correlation of MVD (microvessel density), with clinical and pathological parameters. Six studies were mentioned in their research paper. These experiments elucidated the fact that the higher MVD is directly proportional to the level of tumor differentiation. With regard to the survival terms and recidive occurrence, conclusions of the mentioned above studies show controversial information, suggesting the idea that survival and the recurrence could be influenced by a wide spectrum of factors, including the comorbidities and the pathological predominance of the mechanisms of tumor expansion other than. De la Torre [19], Garcia et al (2006) studied the angiogenic and lymphangiogenic phenotypes of a relatively large but heterogenous cohort of thyroid proliferative lesions (n = 191). Using immunohistochemistry for CD34, lymphatic vessel endothelial receptor-1 (LYVE-1) (specific markers for vascular and lymphatic endothelium respectively), vascular endothelial growth factor (VEGF-A), VEGF-C, this study analyzed MVD, LVD (lymphatic vascular density), and expression of angiogenic and lymphangiogenic factors in normal thyroid (n = 19), multinodular goiter (n = 25), toxic multinodular goiter (n = 8), Graves' hyperplasia (n = 22), follicular adenoma (n = 54), papillary carcinoma (n = 27), incidental papillary microcarcinoma (n = 8), follicular carcinoma (n = 20) and medullary carcinoma (n = 8). MVD was decreased in proliferative lesions, benign and malignant, compared with normal tissue (P < 0.0001). In contrast, VEGF-A expression was increased in major thyroid carcinomas when compared with papillary microcarcinomas, benign lesions and normal gland (P < 0.0001). LVD was higher in PC (papillary cancer) and PMC (papillary microcarcinoma) (P = 0.001), and VEGF-C expression was increased in papillary carcinomas (P < 0.0001). Despite higher LVD and increased expression of VEGF-A and VEGF-C in thyroid cancers, these markers were not related to poor prognosis in terms of tumor size, multifocality and/or presence of lymphatic or distant metastases. In conclusion, angiogenesis is reduced in thyroid proliferative lesions compared with normal thyroid tissue. However, VEGF-A expression is upregulated in thyroid cancers. Lymphangiogenesis and VEGF-C expression are increased in thyroid tumors prone to lymphatic metastases. This may be an important mechanism underlying the differences in metastatic behavior between papillary and follicular thyroid cancer [19].

Jebreel [27] et al (2007) evaluated the relation between VEGF, its receptors (VEGFR-1 and VEGFR-2) and MVD in thyroid diseases. In that study, immunostaining for VEGF and VEGF receptors was performed in 66 specimens of thyroid tissue, comprising 17 multi nodular goiter (MNG), 14 Graves’ disease, 10 follicular adenoma, 8 Hashimoto’s thyroiditis, 7 papillary carcinoma and 10 normal thyroid specimens. Thyrocyte positivity for VEGF and VEGF receptors was scored 0 - 3. Immunohistochemistry for CD31 and CD34 on the same sections was performed to evaluate MVD. Immunohistochemical staining of VEGF in thyrocytes was positive in 92% of all the thyroid tissues studied. Using an immunostaining intensity cut off of 2, increased thyrocyte staining was seen in follicular adenoma specimens, MNG and normal thyroids compared with Hashimoto’s thyroiditis and Graves’ disease (P < 0.05). Similarly, VEGF thyrocyte expression in Graves’ disease was less than other pathologies (P < 0.05). VEGFR-1 expression and the average MVD score did not differ between the different thyroid pathologies. VEGF distribution and expression was lower in the autoimmune pathologies of Graves and Hashimoto’s than those associated with autonomous growth processes. Conversely, both VEGFR-1 and VEGFR-2 were widely expressed in the thyrocytes of both benign and neoplastic thyroid disease, suggesting that up-regulation of VEGF but not its receptors occurring as tissue becomes autonomous. There was no clear relationship between MVD measurement and thyroid pathology [27].

## Angiogenesis in Papillary Thyroid Cancer

Papillary carcinomas have a MVD that is, on average, threefold higher than that present in the peritumoral normal thyroid tissue. Moreover, intratumoral blood vessels have distinctive morphological and immunohistochemical features. In fact, aggregated vascular complexes (glomeruloid structures) have been identified in the stroma of tumor papillae, but not in the normal thyroid or in follicular adenomas or carcinomas. In addition, the oncofetal fibronectin EDB (extra domain B) has been demonstrated in the tumor stroma and in the vascular basement membranes of tumor papillae [28].

The first report demonstrating that the balance between angiogenic and antiangiogenic factors correlates with distinct invasion to other organs and neovascularization of papillary thyroid carcinoma belongs to Tanaka [5] et al (2002). VEGF expression strongly correlated with other angiogenic factors. The cytoplasm of cancer cells stained positive for all factors. Tie-2 and TSP-1 receptor also appeared in endothelia of microvessels. TSP-1 inversely correlated with the degree of invasion of the primary tumor to other adjacent organs and with MVC (microvessel count). A higher MVC correlated with poorer survival. To clarify the balance between angiogenic and antiangiogenic factors in the same tumor, investigators calculated the ratio of each angiogenic factor against TSP-1 as the antiangiogenic factor. The ratios of VEGF/TSP-1, VEGF-C/TSP-1, and Ang-2/TSP-1 (ang = angiopoietins) significantly correlated with a higher MVC. Furthermore, the ratios of VEGF/TSP-1 and Ang-2/TSP-1 significantly correlated with the degree of infiltration. However, in their study, there was no correlation between the clinical data, such as age, gender, tumor size, and the expression levels of angiogenesis-related factors [5].

Akslen [29] and Livolsi (2000) examined a series of 128 papillary carcinomas with respect to MVD and patient survival. Follow-up was obtained for all cases (median, 145 months). Scientists found a mean MVD of 216 per mm^2^ (range, 35-751), and there was an average of 3.14 times more vessels in the tumors, when compared with surrounding non-neoplastic thyroid tissue. MVD was inversely related to age, tumor diameter, histological grade, and primary tumor extent. Furthermore, increasing MVD tended to be associated with improved survival (P = 0.056). Scientists concluded that their data indicate that angiogenesis is important for the development and maintenance of papillary thyroid carcinomas, although it was not identified as a prognostic factor [29].

Scarpino [28] et al (2003) described two types of vessels which are involved in providing vascular support to the papillary thyroid carcinomas: a delicate network of capillary network of capillary vessels located in the stroma of tumor papillae that were weakly stained for EC-NOS (endothelial cell nitroxide synthase); and large venous spaces, sometimes with a muscular coat, located in the peritumoral fibrous tissue. They were intensely stained for EC-NOS, a marker of endothelial activation; some endothelial cells were positive for Ki-67, a marker of proliferation. The venous spaces were often in close contact with infiltrating tumor cell nests and provided images of vascular invasion. In that study, scientists found that papillary carcinoma cells contain RNAs (ribonucleic acid) for VEGF, VEGF-C and angiopoietin, and produce large amounts of VEGF. Moreover, they suggested an idea that, besides tumor cell migration towards blood vessels, VEGF and/or other angiogenic factors released by tumor nests are capable of attracting endothelial cells. According to Scarpino et al (2003), the frequent occurrence of vascular invasion in thyroid tumors might derive from a combination of mutual events involving both migration and attraction of tumor cells and endothelial cells [28].

Another investigation of angiogenesis in thyroid nodular lesions and in papillary cancer in particular was performed by Rzeszutko [30] et al (2004). Using immunohistochemistry, the authors of that study evaluated number of vessels in various nodular lesions of the thyroid (54 cases). Expression of CD34 antigen and MVD were evaluated in sections of archival paraffin blocks originating from the local institutions. MVD was assessed in ten different fields per section in ‘hot spots’. Expression of CD34 was quantified using computerized image analysis and, then, MVC and MVA (microvessel area) were calculated. In thyroid tissue with benign lesions, the MVC (31.7) was higher than in neoplastic lesions (22.3), although no differences in MVA were observed. This observation, according to the opinion of the researchers, points to differences in the size of newly formed vessels in individual nodular lesions of the thyroid [30].

Stabenow [31] et al (2005) based on their experimental results suggested that angiogenesis is more intense among the classic and tall cell variants of metastatic tumors, showing that microvessel count can be an indicator of metastatic potential in these histological subtypes of papillary thyroid carcinoma. In their research, patients that developed recurrent disease had a tendency to exhibit higher angiogenesis; however, there was no association between MVD and prognostic index groups. Finally, authors of that experimental work demonstrated the importance of the study of angiogenesis in papillary thyroid carcinoma.

## Angiogenesis in Follicular Thyroid Tumors

It is assumed that thyroid follicular neoplasms (adenoma and carcinoma) may pose difficulties to the differential diagnosis. Because such a distinction is not possible at FNA (fine needle aspiration), surgery is often performed. Clinical information such as age, sex, and node size is important in case of suspected carcinoma. Follicular carcinoma is characterized by capsular invasion, vascular invasion, and metastatic dissemination mainly by the hematogenic pathway. This invasion depends on collagen degradation in capsule and in subendothelial basement membrane. Collagen degradation has been widely researched in the angiogenesis process and in the hematogenic dissemination mechanism. Frigugliett [32] et al (2000) performed clinical and histopathologic assessment of 74 follicular neoplasms, as well as immunohistochemical reactions for CD-34 protein to estimate angiogenesis and for metalloproteinase-9, an enzyme that degrades type IV collagen. The research was carried out retrospectively in 74 patients who had surgery and were followed up at the local institutions. Clinical, histologic, and immunohistochemical variables were compared among the groups of follicular neoplasms and a control group of 36 patients with colloid goiter. No significant statistical difference was found between patients with follicular adenoma and thyroid follicular carcinoma concerning sex (P = 0.092), age (P = 0.098), thyroid node size (P = 0.426), vascularization (P = 0.388), and immunostaining intensity for metalloproteinase-9 (P = 0.055). The proportion of immunoreactive cells for metalloproteinase-9 in follicular carcinoma cases was higher than that observed in follicular adenoma cases. Patients in more advanced stages of carcinoma were more than 45 years old (P = 0.006), presented extensive invasion (P < 0.001), had less vascularization (P = 0.046), and had a higher proportion of immunoreactive cells for metalloproteinase-9 (P < 0.001). Finally, researchers calculated that the proportion of immunoreactive cells for metalloproteinase-9 in follicular carcinoma was higher than that observed in follicular adenoma, with a significant statistical difference (P < 0.001). Researchers proposed an idea that the described method must be developed to apply in material obtained by FNA to differentiate follicular adenoma from carcinoma [32].

Caroline Kim [33] et al (2007), based on their experiments, suggested that deletion of the pituitary tumor-transforming gene in TRbPV/PV (thyroid receptor beta) mouse thyroids decreases cell proliferation, up-regulates p21, reduces angiogenic factors such as FGF2, and leads to an overall improvement in survival. Their study highlights the fact that pituitary tumor-transforming gene promotes angiogenesis in a mouse model of follicular cancer similar to the tumors localizing elsewhere.

## Angiogenesis in Medullary Thyroid Neoplasm

Medullary thyroid cancers are characterized by the presence of abundant cells, loose groups, poorly defined borders, often multi- or binucleation, eccentrical (plasmacytoid) nuclei, and detection of amyloid [34]. As I could find, no data are available on the description of typical morphological patterns of angiogenesis in medullary carcinomas of the thyroid and only several reports regarding the angiogenic activities in that disease are published.

Zhang [16] et al (2005) in laboratory investigation showed that ASODN (antisense oligonucleotide) can suppress endothelial cell growth and inhibit tumor angiogenesis possibly by specifically blocking VEGF expression in medullary thyroid carcinoma. Bugalho [35] et al (2008) measured VEGF levels in medullary cancer patients. Researchers concluded that serum VEGF levels in medullary thyroid cancer patients are not significantly different from those found in healthy patients and did not correlate with the extension of disease. Thus, the serum VEGF levels in medullary thyroid cancer patients do not appear useful to select potential candidates for therapies with tyrosine kinase inhibitors. Failure to demonstrate high levels of serum VEGF in medullary thyroid cancer patients with distant metastases, in contrast to what happens in patients with differentiated thyroid cancer, suggests the involvement of other proangiogenic factors. Petrangolini et al (2006) tested the antitumor activity of RPI-1 (the indolinone RET tyrosinase kinase inhibitor) against large established subcutaneously thyroid tumor xenograft, a human medullary thyroid carcinoma harboring oncogenic MEN-2A-type RET mutation (MEN = multiply endocrine neoplasia). Oral treatment with RPI-1 caused growth arrest or regression in 81% treated tumors. An additional finding in that study was the significant reduction of MVD in thyroid tumors from mice receiving RPI-1 treatment. Although a direct inhibitory effect on endothelial cells cannot be ruled out, an indirect antiangiogenic effect could be related to inhibition of the oncogene-dependent angiogenic phenotype in light of the marked inhibitory effect of RPI-1 in VEGF expression/secretion by the thyroid tumor cells. Such property is of particular relevance considering that VEGF secretion has been found constitutively activated in some thyroid cancers including medullary thyroid cancers. Even the loss of cellularity observed in treated tumors might be at least in part of the consequence of tumor hypoxia.

## Angiogenesis in Anaplastic Thyroid Cancer

The expression of VEGF *in vitro* has been shown to correlate with *in vivo* aggressiveness of the tumors, with anaplastic tumors having the greatest level of expression of VEGF [18].

Xu [6] et al (2001) investigated the anticancer effects of combined manumycin (a farnesyltransferase inhibitor) and paclitaxel (a microtubule inhibitor) against anaplastic thyroid carcinoma. Scientists presented data showing that the combination of paclitaxel (found in the bark of the Pacific yew tree, is an inhibitor of microtubule function) and manumycin (a natural product of Streptomyces parvulus, inhibits farnesyltransferase by competing with the farnesyl pyrophosphate substrate) provides improved antineoplastic activity *in vivo* without increased toxicity. They observed that the tumor xenografts that were treated with manumycin were paler than those not exposed to manumycin. A hypothesis that can explain this observation is that manumycin inhibits angiogenesis. Researchers concluded that manumycin plus paclitaxel is an effective combination against anaplastic thyroid carcinoma, and inhibition of angiogenesis plays a role in the antineoplastic effect of this combination [6].

A large percentage of anaplastic thyroid carcinomas have been shown to harbor the V600E B-Raf point mutation, leading to the constitutive activation of the mitogen-activated protein kinase pathway. Anaplastic thyroid carcinoma’s invasion, metastasis, and angiogenesis are in part dependent on the gelatinase class of MPMP (matrix metalloproteinases). The explicit targeting of these two tumor markers may provide a novel therapeutic strategy for the treatment of anaplastic thyroid carcinomas. The MMP-activated anthrax LeTx (lethal toxin), a novel recombinant protein toxin combination, shows potent mitogen-activated protein kinase pathway inhibition in gelatinase-expressing V600E B-Raf tumor cells *in vitro*. Based on the literature data and their own experimental work, Alfano [36] et al suggested that the MMP-activated LeTx could be used not only in the clinical management of V600E B-Raf ATC (anaplastic thyroid cancer) but potentially in any solid tumor.

Kim [33] et al (2007) showed that Sorafenib, a multikinase inhibitor of the B-raf, VEGF receptor-2, and platelet-derived growth factor receptor beta kinase exert significant antitumor activity in an orthotopic xenograft model of anaplastic thyroid neoplasias via a potent antiangiogenic effect. Recently, Zhu [37], Wenbo et al (2009) in the experimental research showed that triptolide, a small molecule from a Chinese herb, may function as inhibitor of tumor angiogenesis and invasion and may provide novel mechanistic insights into the potential therapy for human anaplastic thyroid cancers.

Therefore, literature data from the mentioned sources show that the majority of studies investigating angiogenesis in anaplastic thyroid cancer are pharmacology-oriented.

## Lymphangiogenesis in Malignant Thyroid Tumors

**Figure 4:**
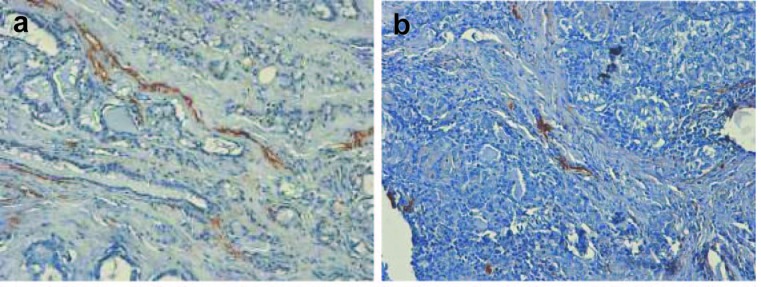
(a, b). Classical examples of papillary thyroid cancer, stained with hematoxylin and eosin and D2-40 marker. Lymphatic vessels are brown-colored (x 300).

Publications on the lymphangiogenesis in thyroid are scarce. The driving force of scientific progress in this field is recent advent of lymphatic vessel markers; among them is D2-40, which was applied in our study (Fig. 4). D2-40 antibody originally reacts with O-linked sialoglycoprotein, expressed on lymphatic endothelium, fetal testis, and testicular germ tumors. It was assumed that tumors can induce lymphangiogenesis and the growth of lymphatic vessels, and the major factors for these processes are vascular endothelial growth factors (C, D, A in order of significance). Several questions remain unresolved, relating to the mechanisms by which expression of VEGF is increased in primary tumors, and non-lymphangiogenic functions of these molecules in promoting lymph node metastases may exist. These functions are the activation of existing lymphatic endothelium causing an increase of size with the production of mitogenic or chemotactic factors or alterations in lymphatic endothelial-tumor cell adhesion, sufficient to increase the lymphogenic spreading [38, 39]. Lymph node lymphangiogenesis facilitates the metastatic seeding by tumor cells which in turn further promote lymphatic vessel growth, thereby promoting metastasis to non-sentinel lymphatic nodes and via the thoracic duct to the blood vasculature and distant organs. The major laboratory techniques for the investigation of lymphangiogenesis are immunochemistry and RNA-based assays. Hall [40] et al (2003) were first who investigated the location and morphologic characteristics of tumor lymphatics in patients with papillary carcinomas of thyroid. With the use of LYVE-1 as the marker, Chalkley counting as the vessel calculation procedure, a high LVD was associated with the presence of regional lymph node metastases. However, the presence of intratumoral lymphatics was not a significant predictor of tumor recurrence (P = 0.42, log-rank test).

Interestingly, research of Cheong [41] et al (2010) showed no significant differences in lymphatic and blood vessel density of papillary thyroid carcinomas and microcarcinomas (less than 1 cm in size). However, there was a significantly higher LVD in patients older than 45 years old (more apparent in the PTC group) and multicentricity with extrathyroidal extension. Multicentricity was defined as the presence of additional tumor foci noncontiguous with the primary tumor. Extrathyroidal extension was defined as the extension of the tumor beyond the capsule of the perithyroidal soft tissue.

## Conclusions

The current data from the conducted studies are insufficient to draw valid conclusions regarding the power of correlation between the MVD and pathological and clinical parameters. The major limitations of the conducted studies are the minor sample numbers, differences among methods applied (for example, Chalkley and computer-assisted MVD calculation) and the lack of research data concerning correlative analysis of a comprehensive set of values including the comorbidities, inheritance patterns, exposure to toxic substances, and so on.

Future studies should be performed in order to clarify the differences between angiogenic patterns in various histological types of thyroid neoplasm stained with a wide armamentarium of immunochemical substances, not only with conventional stains (CD34, CD31, F8 and so on). Though VEGF is the major proangiogenic factor in tumors and in benign disease and normal tissues, new angiogenesis markers should be investigated for a better recognition of differences of the pathological entities.

The other idea is to investigate the effect of ionizing radiation and carcinogens on vascular structures observed in thyroid tissues. That research might elucidate the pathological changes similar to those, which occur in nuclear atomic disaster survivors and provide knowledge for the implementation of drugs for radiation damage prevention and cancer treatment. Novel research opportunities are provided by the recent advances in digital pathology and image processing. Various parameters derived via the image analysis can be correlated with the available clinical and laboratory information in order to discover new mechanisms, correlative chains, pathways and predictors.

## Dedication

Dedicated to professor Leontuk, who agreed to become my PhD thesis Mentor just a few months before his death.
